# Health technology assessment for sexual reproductive health and rights benefits package design in sub-Saharan Africa: A scoping review of evidence-informed deliberative processes

**DOI:** 10.1371/journal.pone.0306042

**Published:** 2024-06-27

**Authors:** Warren Mukelabai Simangolwa, Josue Mbonigaba, Kaymarlin Govender

**Affiliations:** 1 Health Economics and HIV/AIDs Research Division, University of KwaZulu Natal, Durban, South Africa; 2 College of Law and Management Sciences, University of KwaZulu Natal, Durban, South Africa; PLOS: Public Library of Science, UNITED KINGDOM

## Abstract

**Background:**

Health technology assessment uses a multidisciplinary approach to support health benefits package design towards universal health coverage. The evidence-informed deliberative process framework has been used alongside Health technology assessment to enhance stakeholder participation and deliberations in health benefits package design. Applying the evidence-informed deliberative framework for Health assessment could support the morally diverse sexual reproductive health and rights (SRHR) benefits package design process. However, evidence on participation and deliberations for stakeholders in health technology assessment for SRHR benefits package design has not been curated in sub-Saharan Africa. This study synthesises literature to fill this gap.

**Methods:**

This scoping review applies the preferred reporting items for systematic reviews and meta-analyses extension for scoping reviews, and deductive analysis following the evidence-informed deliberative processes framework. The search strategy uses the Guttmacher–Lancet Commission-proposed comprehensive definition of SRHR and the World Health Organisation’s universal health coverage compendium of SRHR interventions to generate search terms. Six databases and biographical hand searches were used to identify studies in Sub-Saharan Africa from 1994.

**Results:**

A total of 14 studies met the inclusion criteria. Evidence for yearly public budgets and explicit SRHR health technology assessment processes was not found. In 12 of the studies reviewed, new advisory committees were set up specifically for health technology assessment for SRHR priority-setting and benefits package design. In all decision-making processes reviewed, the committee member roles, participation and deliberations processes, and stakeholder veto powers were not clearly defined. Patients, the public, and producers of health technology were often excluded in the health technology assessment for the SRHR benefits package design. Most health technology assessment processes identified at least one decision-making criterion but failed to use this in their selection and appraisal stages for SRHR benefits design. The identification, selection, and scoping stages in health technology assessment for SRHR were non-existent in most studies. In 11 of the 14 processes of the included studies, stakeholders were dissatisfied with the health policy recommendation from the appraisal process in health technology assessment. Perceived benefits for evidence-informed deliberative processes included increased stakeholder engagement and fairness in decision-making.

**Conclusion:**

To support the integration of diverse social values in health technology assessment for fairer SRHR benefits package design, evidence from this review suggests the need to institutionalise health technology assessment, establish prioritisation decision criteria, involve all relevant stakeholders, and standardise the process and assessment methodological approaches.

## Introduction

Universal health coverage requires everyone to access the quality health services they need without experiencing financial hardship [1]. The development of a health benefits package is central to universal health coverage policy [2]. The health benefits package creates explicit entitlements for the public by prioritizing interventions within resource constraints [3]. Defining an effective yet affordable health benefits package avoids having aspirational "de jure" services that, while available in theory, differ from the "de facto" set of interventions accessed [3]. The program of action for the 1994 Cairo International Conference on Population and Development defined and proposed a package for universal access to sexual and reproductive health services and reproductive rights [4]. In Sub-Saharan Africa, the sexual reproductive health and rights (SRHR) 2005 continental policy framework [5] and its operationalization through the 2016–2030 Maputo action plan [6], reinforced the Cairo program of action aspirations in line with the 2030 sustainable development goals and universal health coverage objectives.

To accelerate global progress towards the program of action aspirations, the 2018 Guttmacher–Lancet Commission proposed that each country adopt the comprehensive definition of SRHR interventions into a health benefits package that should be universally available [7]. The Guttmacher–Lancet Commission’s definition is consistent with the World Health Organisation’s proposed SRHR benefits package [8, 9]. The Guttmacher–Lancet Commission interventions are cost-effective and affordable for most low and middle-income countries [7]. However, health benefits packages in most low and middle-income countries, only include pregnancy-related care, family planning, and HIV/AIDS, from the proposed Guttmacher–Lancet Commission’s SRHR definition. The other Guttmacher–Lancet Commission interventions, notably services on reproductive cancers, infertility, intimate partner and sexual violence, abortion care, comprehensive sexuality education, and sexual health, are seldom included [7, 10]. In most countries, where SRHR services are included, difficulties in public finance, delivery, quality, and access for vulnerable and marginalised populations are widespread [7]. The limited delivery and outright exclusion of some SRHR services in a health benefits package are likely to infringe on people’s right to health, ultimately threatening a country’s UHC objectives [7]. The High-Level Commission on the International Conference on Population and Development 25 years follow-up, identified Sub-Saharan Africa as having made the slowest progress in the world in terms of effective, accelerated implementation and funding of the program of action for the comprehensive SRHR package [11]. Trade-offs are inevitable in SRHR benefits package priority-setting and design and as such the Guttmacher–Lancet Commission recommends transparent and participatory approaches to enhance accountability and legitimacy [7].

To encourage the practical application of evidence-informed, transparent, and inclusive decision-making for benefits package design for universal health coverage, the World Health Assembly also passed Resolution WHA 67.23 on health intervention and technology assessment [12]. Health technology assessment is defined as a "multidisciplinary process that uses explicit methods to determine the value of health technology at different points in its lifecycle. The purpose is to inform decision-making to promote an equitable, efficient, and high-quality health system" [13]. There is increasing use of Health technology assessment to support decision-making for health benefits package design in low and middle-income countries [14]. A growing number of low and middle-income countries are using evidence-informed deliberative processes to advance the use of Health technology assessment for health benefits revision and design [15]. It provides practical steps to legitimize health technology assessment decision-making using six steps: (1) installing an advisory committee, (2) defining decision criteria, (3) identification and selection of health technology, (4) scoping, assessment, and appraisal of evidence, (5) communication and appeal, and (6) monitoring and evaluation [15]. The evidence-informed deliberative processes framework is a practical interpretation of the accountability for reasonableness theory. The accountability for reasonableness theory is a procedural framework on conditions for fair decision-making based on four principles: consideration of all relevant values, transparency, provision of appeal processes and ensuring that all these conditions are met [16].

The evidence-informed deliberative processes framework has been used in countries such as Ghana, Moldova, Pakistan, and Ukraine to support explicit priority-setting processes to define their health benefits design initiatives [15]. In Tanzania, components of the evidence-informed deliberative processes framework were used to elicit the capacity needs of the health technology assessment committee to support universal health coverage policy [17]. The Republic of Kazakhstan applied the evidence-informed deliberative process to prioritise 25 health technologies including HIV in the development of a health benefits package for universal health coverage [18]. Similarly, Indonesia utilised the framework for the prioritisation of HIV control interventions [19]. In addition, the Islamic Republic of Iran applied the framework for the inclusion and appraisal of nine multiple sclerosis services for health insurance benefits package revision [20].

The benefits of applying transparent and deliberative processes for health technology assessment to support priority-setting and health benefits package design include improved evidence availability [19], stakeholder involvement [19], and legitimacy of the decision-making process [20]. In Malaysia for instance, the involvement of diverse stakeholders in SRHR priority-setting for universal health coverage, increased understanding and buy-in [21], whereas the lack of involvement in Afghanistan, was perceived to favour donor priorities and donor evidence [22]. In Thailand’s introduction of universal health coverage, the success of SRHR integration into the benefits package has been attributed to coordinated government efforts to use stakeholder-acceptable evidence to extend health services to clients and reduce financial barriers to health [23]. In sub-Saharan Africa, two accessible studies have explored the inclusion of Guttmacher–Lancet Commission proposed SRHR interventions in health benefits packages. In the first research, the authors analysed decision-making processes for SRHR and mapped the Guttmacher–Lancet Commission package across health benefits packages of six countries [24]. The authors of the second study expanded the assessment of the Guttmacher–Lancet Commission SRHR package in a health benefits package to thirty-three sub-Saharan African countries and other low-and middle-income [10]. In both studies, the pattern of SRHR intervention inclusion in health benefits packages, as well as the recommendation for stakeholder participation and deliberations, were comparable to those established by the Guttmacher–Lancet Commission.

Recommendations for the adoption of the Guttmacher–Lancet Commission SRHR package and the use of the evidence-informed deliberative processes framework are recent. However, applying the evidence-informed deliberative processes principles on stakeholder participation and deliberations to current and historic decision-making for the Guttmacher–Lancet Commission SRHR proposed interventions, has the potential to provide a forward-looking model to improve legitimacy and accountability. This scoping review aims to synthesise this evidence by mapping literature in sub-Saharan Africa on the use of health technology assessment (in particular assessment and appraisal) of SRHR health technologies for SRHR priority-setting and benefits package design. It describes the policy context of health technology assessment and characterises the evidence-informed deliberative processes principles (stakeholder involvement, use of an explicit set of decision criteria, use of evidence, transparency) and steps (identification and selection, scoping, assessment and appraisal, communication, appeal, monitoring, and evaluation) for SRHR priority-setting and benefits package design in sub-Saharan Africa.

## Materials and methods

### Design

This scoping review followed the Joanna Briggs Institute (JBI) Manual for Evidence Synthesis [25] and the framework suggested by Arksey and O’Malley [26]. Scoping reviews aim to assess the form and extent of research literature and gaps, to influence policy and practice [27]. Following the EDP six steps described in the background, the study team created a chartering form to collate and summarise evidence from included studies. This scoping review was reported using the Preferred Reporting Items for Systematic Reviews and Meta-Analyses extension for Scoping Reviews [28]; S1 Checklist in S1 File. The scoping review technique was used in this study because it characterises the use of evidence-informed deliberative processes for health technology assessment for SRHR priority-setting and benefits package design in sub-Saharan Africa. The study ethics approval was obtained from ERES Converge in Zambia and the Humanities and Social Science Research Ethics Committee at the University of KwaZulu Natal in South Africa, with approval numbers Apr-2021-007 and REC00004520/2022. A protocol for this scoping review was not registered on a publicly accessible platform but is available upon request from the corresponding author. Given that this paper is based on the review of published literature, no participants were interviewed in the study and therefore no informed consent was needed.

### Eligibility criteria

Original country-specific studies were included if they described health technology assessment for benefits package design processes for public resource allocation in sub-Saharan Africa, across SRHR program-specific interventions, or the SRHR package within a health benefits package. Inclusion was restricted to English studies between 1994 and 2022 to coincide with the initiation of the International Conference on Population and Development. Studies for the humanitarian "Minimum Initial Service Package" for SRHR were excluded due to the uniqueness of decision-making in emergency setups. Studies that merely sought to develop decision-making criteria, model priority-setting procedures, or concentrate on privately sponsored insurance plans or funding mechanisms were also not included as these did not describe actual public resource allocations for SRHR.

### Literature search

We searched PubMed, Medline, Scopus, Web of Science, Science Direct, and PsycInfo and search results for the first 200 studies in Google Scholar. We used the Guttmacher–Lancet Commission SRHR comprehensive definition to identify interventions [7] and the detailed world health organisation’s universal health coverage compendium version 1.2 for explicit actions for each intervention [9, 29]. S2 File contains the search terms and a PubMed electronic search strategy. We also searched bibliographies for included studies and specific websites for institutions active in SRHR programming and research (S2 and S3 Files). All searches were performed in August 2022.

### Study selection and analysis

**Fig 1:**
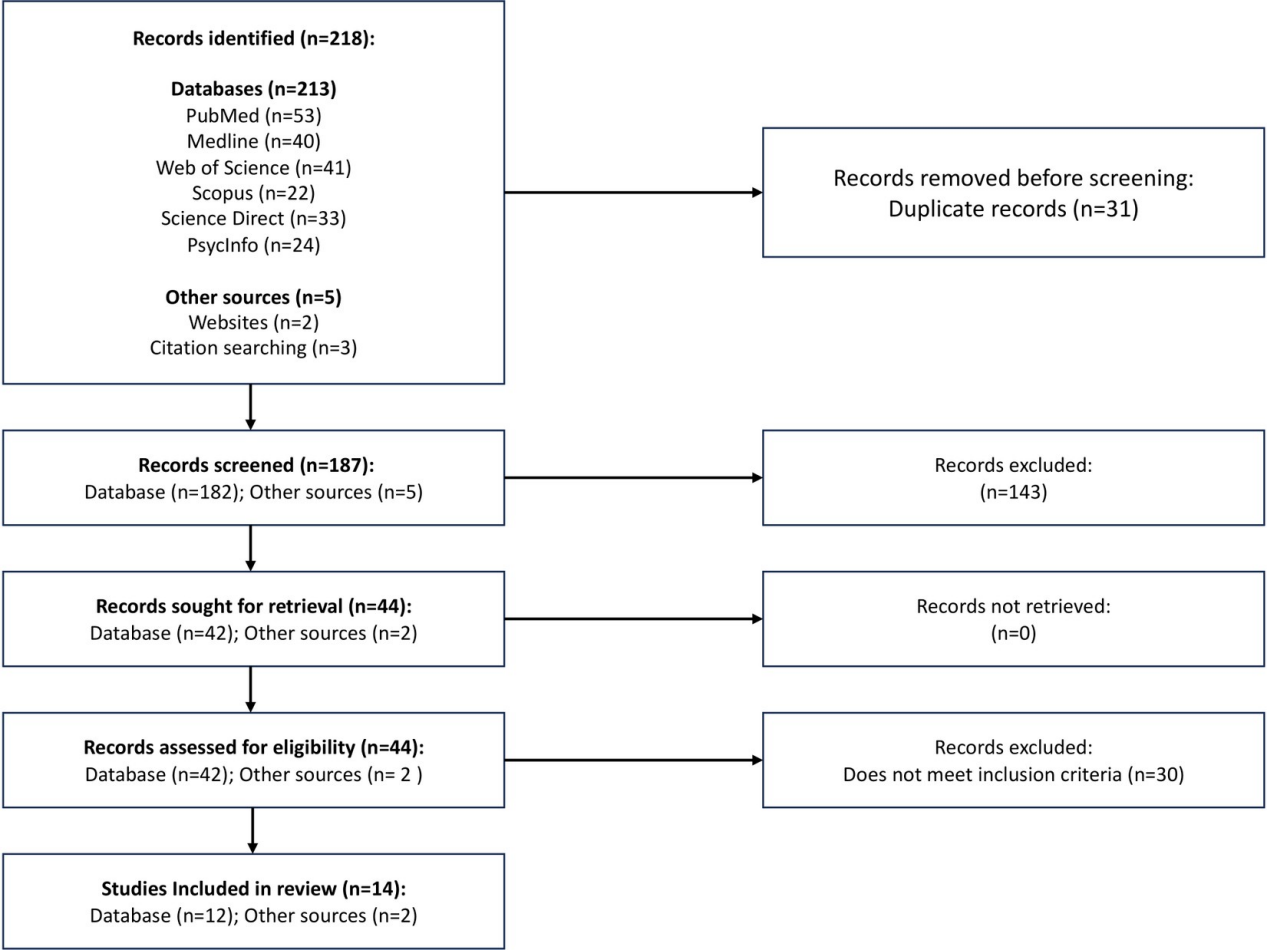
Study selection process flowchart.

All duplicates were removed, and WS double-screened prospective papers by title and abstract. WS and JM conducted a full-text screening of the remaining studies. In cases of inclusion uncertainty, KG independently reviewed each case using the deciding criteria that a study should document actual resource allocation decision-making for SRHR. All included studies were imported to Zotero-6.0.13 for reference management. Fig 1 shows the Preferred Reporting Items for Systematic Reviews and Meta-Analyses extension for Scoping Reviews flow chart, for the search and inclusion process [30]. Deductive thematic analysis responding to the evidence-informed deliberative processes framework was used in the analysis. We did not perform any quality assessments on the included studies because the scoping review was exploratory.

## Results

The flow chart in Fig 1 summarizes the study selection process. A total of 218 studies were identified; 213 from 6 databases and 5 from other sources. There were 31 duplicate studies removed, leaving 187 studies for screening. An additional 143 studies were excluded based on the title and abstract. Of the 44 remaining studies, 30 did not match the selection criteria and were removed after a full screening. As a result, 14 papers were included in the review: 12 from database peer-reviewed searches and 2 from manual searches of grey literature.

### Characteristics of included studies

The characteristics of the included studies are shown in the S4 Table in S4 File. Half of the studies focused on decision-making for the whole sexual reproductive health and rights package within a health benefits package, while the other half examined SRHR program-specific interventions. Three (03) of the 7 studies on program-specific intervention categories focused on human immunodeficiency virus / acquired immunodeficiency syndrome, while the other 2 focused on abortion, cervical cancer, and family planning. Ipas, World Health Organisation, United Nations Development Programme, United Nations Population Fund, World Bank, United Nations Children’s Fund, World Food Program, and the United States Agency for International Development, were among the development assistance partners who supported the priority-setting and benefits package design processes. Others were the Department for International Development, the Vaccine Alliance, the President’s Emergency Plan For Aids Relief, the European Union, the Global Fund, the Global Financing Facility, and the Partnership for Maternal Newborn and Child Health. Zambia, Malawi, Tanzania, and Ethiopia each had 2 studies among the 14 included. Ghana, Uganda, South Sudan, Kenya, Botswana, and South Africa each had 1 study.

The country for the institution of affiliation for each author was used to determine authorship status. In cases when an author had more than one institution of affiliation, the SSA institution was given precedence. In SRHR, the position of first and last authorship has been associated with research leadership, success in capacity building, and balance in research collaborations [31]. One study was not included in the authorship analysis due to insufficient authorship details. North America and Europe had 2 and 4 first authors. In Africa, Ethiopia, and Tanzania each had 2 first authors, and Kenya, South Africa, and Botswana each had 1. For the last authorship, all authors not in sub-Saharan Africa, were based in Europe, namely: Norway (2 authors), Sweden, the Netherlands, and the United Kingdom. Sub-Saharan African authors represented institutions from Kenya, Botswana, South Africa, Tanzania, Uganda, Ghana, Malawi, and Zambia.

#### Health technology assessment policy and contextual factors

The legitimacy of priority-setting and benefits package design in a society with diverse social values necessitates an awareness of the policy context. All 14 included studies did not report the existence of a formal decision-making body and an explicit guiding policy statement supporting the use of health technology assessment.

Across all the 14 studies, there was no mention of yearly public dedicated health technology assessment budgets. All reported SRHR priority-setting and benefits package design processes were primarily funded by donors [32–36]. The World Health Organisation was the most common development assistance partner, financing 7 of the 14 processes. Four (4) of the 14 SRHR priority-setting and benefits design processes were supported by the Global Fund, while 3 were supported by the United States Agency for International Development, the World Bank, and United Nations Population Fund. Two (2) processes were supported by Ipas, the President’s Emergency Plan For Aids Relief, the Vaccine Alliance, United Nations Children’s Fund, and the Global Financing Facility, while one 1 was supported by Swedish International Development Cooperation Agency, Department for International Development, the World Food Programme, the European Union, and the Partnership for Maternal Newborn and Child Health. Development assistance partners tended to have more control over resource allocation decisions for activities they funded [34–36]. For example, Department for International Development set the strategic priority-setting and benefits design objectives for the health pooled fund in South Sudan on behalf of the Ministry of Health and a consortium of development assistance partners [36]. Furthermore, no in-country process guidelines for public priority-setting for benefits package design were referred to. The essential drug list decision-making processes in South Africa were identified as having better-defined procedural guidelines than the health benefits package design process [37]. In addition, guidelines on economic evaluations, or other dimensions of value approach for health technology assessment for SRHR and broadly health benefits package design, were not available. In some cases, historical processes indicated the use of criteria such as cost-effectiveness [33, 34, 36, 38], the burden of disease [34, 38, 39] and affordability [35], however, these did not follow any existing country guidelines.

Technical support for decision-making was provided mainly through development assistance partners, health technology assessment networks, and academia. These included the University of York, the University of Bergen, Harvard T.H. Chan, and the International Decision Support Initiative [35, 40, 41]. Across the 14 included studies, only 1 lacked interdisciplinary teams with an understanding of health technology assessment techniques and connections to regional networks [36]. Despite the existence of multidisciplinary teams, the application of health technology assessment to inform decision-making was limited across all studies. In Malawi, supplementary multidisciplinary training was conducted before a decision-making process to provide additional mentorship [33]. The limited availability of skills to perform economic evaluations resulted in historical decision-making processes using less methodologically robust alternative measures [37, 38, 41].

#### Installation of advisory committee

In an ideal health technology assessment process, an advisory committee represents the interest of all diverse stakeholders in the decisions made [15]. As a result, the involvement of stakeholders, the justification for their inclusion, their roles, and the quality of the deliberations are crucial in informing the validity of the health technology assessment process. From the included studies, 12 new advisory committees were established to specifically support SRHR priority-setting and benefits package design, whilst 2 were already existing [34, 36]. In 13 studies, the Ministry of Health created advisory committees, whereas 1 advisory committee was initiated by a health sector funding consortium [36]. In as much as the Ministry of Health appointed stakeholders to the advisory committees, setting these up was strongly driven by development assistance partners [32, 33, 35, 36, 42]. The advisory committee’s names were Assessment Team [32, 36], Committee [37, 38, 40], Technical Working Group [35, 41], Advisory Panel [43], Commission [34, 44], Management Team [39, 44], and Funding Team [36]. Across the 14 studies, there was no public advertisement for advisory committee roles. Only one study suggested that certain stakeholders were included because they represented distinct social values [32]. The inclusion of stakeholders across the evidence-informed deliberative processes recommended 7P’s: patients and public, policymakers, providers, payers, purchasers, principal investigator, and product producers, varied. No process included patients directly, whereas only 4 studies included public representatives [32, 36, 37, 44]. Policymakers were included across all studies, through Ministries of Health, Social Welfare, Local Government, Gender, Education, Finance, and Labour and Social Protection officials. The inclusion of health technology purchasers was not indicated, and only the processes identified by the studies in Zambia and South Sudan on priority-setting for HIV/AIDS and the entire sexual and reproductive health rights, included payers [36, 40]. Except for two, all processes included principal investigators [39, 44]. The inclusion of health technology manufacturers in advisory committees was not reported in the processes for all included studies.

The roles and responsibilities of stakeholders for the advisory committee were associated with implementing the final advisory committee decisions and not the legitimacy of the process [32, 33, 38]. None of the included studies described veto powers for stakeholders and whether the participation of stakeholders was formal or informal. In 1 study, whereas the advisory committee was reported to be inclusive, evidence showed limited involvement of the recruited stakeholders [39]. The participation of other stakeholders, not a part of the advisory committee, was through consultation [34], communication [32, 33, 40], and participation in implementation [32, 33, 39, 44].

#### Decision criteria

The health system’s goals of achieving universal health coverage, equity for the worse-off, and health benefits maximizing are important sources of social values [15]. However, these social values must be operationalized into decision criteria to be useful in priority-setting and benefits package design. In all SRHR processes under consideration, only 3 studies did not indicate the decision criterion preferred for their decision-making. The decision criterion preferred for use in benefits package design for the included studies was, quality [38], safety [43], the severity of disease [43], public acceptability [41], alignment to national and international priorities [34, 39], effectiveness [32, 38, 43], political acceptability [34, 35, 37, 42], programmatic constraints [34, 35, 37, 43], and financial risk protection [39, 41, 43, 45]. Others are budget impact [34, 37, 40–42], equity [34–36, 41, 43, 45], cost-effectiveness [34–38, 41, 45], and disease burden [32, 35, 37, 39–43].

Elicitation approaches for decision criteria were a review of national health policy and strategic documents [41], consultation with experts [35, 41], and modified nominal group technique [43]. As an example of consultation with experts, the process described in a study by Eregata and others, in Ethiopia, held 10 consultations and deliberative meetings with local and global experts [41].

#### Health technology identification and selection

A comprehensive inventory listing of available interventions using health sector strategic plans, program strategies, publications, and reviews from the World Health Organisation’s Universal Health Coverage and Disease Control Priorities Three Compendium, was used to identify health technology [41, 45]. Other approaches included pre-identification of health technologies following research and stakeholder engagement [32, 33], and development assistance partners identifying health technologies aligned with their plans [36, 38]. Additionally, existing benefits packages were used to map health technology [39, 40]. Once identified, most studies did not indicate how the health technologies were selected for the assessment stage. Only 3 studies indicated the use of the nomination process [32, 36, 41].

#### Scoping for health technology

Scoping in health technology assessment involves setting explicit objectives for the assessment phase. Only five studies had some form of the scoping process. Objectives were set by stakeholders based on the identified criteria [41], in others, the resource allocation tools had predetermined objectives [32, 33, 35, 36]. For example, in the case of South Sudan, the Department for International Development’s business case, a tool developed by donors, predetermined objectives based on cost-effectiveness, equity, coverage, and health system stabilisation [36]. The only identified method for scoping was in the process described by Eregata and others, in Ethiopia, which expanded the population, intervention, comparator and outcomes framework, to include payment mechanisms and level of healthcare delivery [41].

#### Assessment of health technology

In the evidence-informed deliberative processes framework, evidence collection and synthesis inform the assessment stage. Across included studies, assessment guidelines included the World Health Organisation’s strategic approach to strengthening sexual and reproductive health policies and programs, Department for International Development’s business case [36], World Health Organisation’s burden of disease measure and World Bank cost efficiency measures [38], and framework for the HIV planning and budgeting process [37]. Others were the World Health Organisation choosing Interventions that are cost-effective for generalized cost-effectiveness analysis, the consolidated health economic evaluation reporting standards checklist for transferability, and the Delphi approach for financial risk protection and equity assessments [41]. Additionally, the one health tool [41, 45], and a cost-effectiveness analysis framework [34, 35] were also used. The Malawi case, for example, ranked interventions using economic evaluation evidence and allowed the consultative process to take account of a wider set of political, ethical and health system considerations. Only about 35% of interventions under assessment had sufficient data on disease burden, efficacy or cost to support decision considerations [35]. Results from the assessment stage were presented to advisory committees [32, 36, 37, 39, 45] and wider stakeholders [33–35, 41].

#### Appraisal of health technology

The appraisal stage develops recommendations for reimbursement and health benefits package implementation for the included health technology. During the appraisal stage for the reviewed studies, recommendations were generated within the advisory committee alone [32, 35, 36, 38–40, 43–45], shared with broader stakeholders for iterative reflections [33, 41], and print media [34], for wider public communication. However, some processes did not have an explicit appraisal stage [36]. In others, there was a mismatch between the criterion desired for use in decision-making, the evidence generated for assessment, and the evidence presented for appraisal and the criterion used for decision-making [34, 35, 40]. For example, in a process from South Africa, described by Murphy and others, there were tensions between the treasury and decision-makers on the methods used as the former preferred return on investment to budget impact and cost-effectiveness frameworks [37]. In other decision-making processes, evidence on cost-effectiveness analysis and disease burden was unavailable, and the available mortality and morbidity estimates were used [38, 42]. In some appraisal stages, interventions were included because they aligned with the funding partner and Ministry of Health interests [34–36, 39].

The specific roles of stakeholders in the appraisal process were not documented across processes reported by all included studies. The appraisal stage was thought to be highly technical, so the public and other non-technical stakeholders were left out [34, 38]. Tools used for appraisals were multi-criteria decision analysis [41], incremental cost-effectiveness ratio threshold, equity, and financial risk protection score [45]. The remaining 12 studies did not indicate the tools used for the appraisal stage.

#### Communication and appeal

A communication and appeal process provides opportunities to improve the legitimacy of the decision-making process. In all 14 included studies, recommendations from the appraisal process were implemented almost immediately with no appeal provisions. In 11 of the included studies, key stakeholders were dissatisfied with the health policy recommendation from the appraisal process [32–40, 43, 44]. In Ghana, for example, there was tension at the selection of HIV treatment scaling-up interventions, as opposed to other less costly and urgent sexual reproductive programs. However, the lack of appeal processes limited stakeholder opportunities to communicate their displeasure [38].

#### Monitoring and evaluation

The monitoring and evaluation of the evidence-informed deliberative framework evaluates the legitimacy and impact of the health technology assessment recommendations. In all processes for the included studies, the priority-setting and benefits package design did not deliberately embed an evaluation of the legitimacy of the decision-making process. Some reported benefits of using a systematic decision-making process included increased political will [32, 33], increased deliberation between stakeholders [32, 42], increased value for the role of social values by stakeholders [32, 34–37, 39, 43], communication [34, 44], and transparency [35, 44].

The outputs, outcomes, and impacts of health technology assessment included an additional inclusion of a complimentary service [32], health force strengthening [32], improved health service delivery [35, 44], and increased funding [37]. Some challenges were the exclusion of health technology in the benefits package [33], lack of equity in implementation [34, 43], the mismatch between clinical guidelines and essential medicine list and quality of delivery at facility level [35], and lack of consideration of culture values [34].

## Discussion

Sub-Saharan African countries are increasingly implementing health benefits packages to support public resource allocation across health services, including SRHR. However, there is a paucity of information on the inclusion of diverse stakeholders and the quality of decision-making processes to support health technology assessment for a benefits package design for SRHR. This review sought to characterise evidence using the evidence-informed deliberative processes framework in health technology assessment to support decision-making for SRHR program-specific interventions and their inclusion in health benefits packages in sub-Saharan Africa. As this scoping review findings have shown, the environment to support SRHR and broad health benefits package decision-making in sub-Saharan Africa lacks formal health technology assessment institutions, funding, and explicit country policy statements encouraging the use of health technology assessment. Fairer decision-making for SRHR is lacking. Advisory committees to support decision-making do not have clear roles and responsibilities to increase the legitimacy of their decision-making recommendations. Furthermore, the review found that stakeholders included in these committees do not represent diverse social values, including those of the public, patients, industry, and payers. Whereas there are attempts to define decision criteria, there is limited evidence on its use in the assessment and appraisal of SRHR health technology. Additionally, stakeholders were extensively dissatisfied with health technology assessment for SRHR recommendations. The implications of this review’s findings are important to all stakeholders interested in ensuring that decision-making for SRHR accounts for diverse social values fairly and transparently.

To begin, the review has identified challenges in the policy context for sub-Saharan African countries with implications on the use of health technology assessment for SRHR priority-setting and benefits package decision-making, such as budgets, institutional ability to perform health technology assessment, and its use. A recent study by Hollingworth and others exploring health technology assessment institutionalisation in low-and middle-income countries also found that activities are uncoordinated and disconnected from policy [46]. The authors characterised the challenges as poor awareness of the relevance of health technology assessment to assist decision-making, as well as limited health technology assessment institutional and regulatory development, which was worsened by low health technology assessment skill levels and insufficient use of evidence for policy. Similar evidence was also synthesised elsewhere [14, 47]. According to Falkowski and others, a lack of specific health technology assessment units within low and middle-income countries governments contributes to the low financing for health technology assessment initiatives [46]. The foregoing raises a need for guidance to support health technology assessment [48]. Furthermore, in studies identified and included by this scoping review, the Ministry of Health initiated the creation of advisory committees. However, the reasons for the process, inclusion, and roles of specific stakeholders and their veto powers, were unclear. In addition, our study found that SRHR health technology assessment advisory panels mostly comprise researchers, development assistance organizations, and policymakers, while other stakeholders such as patients, the general public, payers, purchasers, and industry are frequently excluded. This worrying trend was also revealed in a recent literature review where authors identified the inclusion of public and vulnerable populations as lacking in priority-setting processes [49]. Their study recommended that priority-setting processes should be explicit on how these marginalised groups should be included and how the participation process should be administered. As encouraged by Kapiriri using the priority-setting case of Uganda, fostering legitimate priority-setting processes requires that government and development assistance partners work together with wider stakeholders to balance decision-making powers [50].

As seen in our review, while most included studies expressed a wish to employ decision criteria for priority-setting, this was seldom implemented. Disease burden, cost-effectiveness, equity, and budget impact were the most sought-after decision criteria, whereas public acceptability, quality disease severity, and safety were the least sought-after. These results closely resemble those identified by Kaur and others [51]. In their research, however, the decision criteria were employed to enhance priority-setting procedures, which was seldom the case in our findings. In our review, there were limited efforts to operationalise the sought-after decision criteria for SRHR decision-making.

The role of development assistance partners in benefits package decision-making is critical. In our study, the identification of SRHR health technologies as potential candidates for selection into the evidence synthesis stage mainly followed the priorities for development assistance partners. As demonstrated by Kapiriri using the instance of Uganda, the perceived role of development assistance partners in the priority-setting process is to direct how their funds should be used for their established priorities [50]. Their research, however, contends that there must be a fair and acceptable balance between national priorities and those of development aid partners [50]. One way to achieve this as shown in our study is through stakeholder capacity development and collaborations using development assistance networks and academia. Countries can leverage decision-support networks such as the international decision-support initiative and the newly formed Health Economics Program at Africa Center for Disease Control, in addition to academia to steer capacity development efforts in sub-Saharan Africa [14].

In our review of the appraisal stage of the studies identified, most data did not correspond to the objectives set at the scoping stage. Whereas there was a measure of tools used for the assessment stage, the use of tools at the appraisal stage was predominantly absent across most studies. These mismatches resulted from data unavailability, lack of clear objectives at the scoping stage, and influence from development assistance partners. For example, the Department for International Development’s business case was used in South Sudan, because the priority-setting process was steered by Department for International Development [36]. Firstly, the unavailability of health and economics data and assessment tools to support health technology assessment priority-setting has been widely discussed elsewhere [14, 46, 47]. This could be because some social value dimensions are difficult to quantify and there are no systems to periodically collect the needed data [46]. Secondly, as shown in the literature, frameworks such as the Integrated health technology assessment for the evaluation of complex technologies, guidance on priority-setting in health care, World Health Organisation’s choosing cost-effective Interventions, and the health technology assessment core model by EUnetHTA could provide additional resources to guide scoping objectives, assessment, and appraisal [47, 52–54].

Appeal processes provide opportunities to improve the quality of the decision-making process [15]. Across all included studies, there were no available avenues for the appeal of the SRHR priority-setting decisions. In fact, in 11 of the 14 included studies, stakeholders were unhappy with the recommendations. As an example of why appeals are important, although not in sub-Saharan Africa, the re-appeals at the National Institute of Health and Care Excellence in the United Kingdom, have a 54% favourable outcome for organizations objecting to an initial decision [55].

In our study, there was no evidence of deliberate use of monitoring and evaluation for SRHR health technology assessment. However, there were benefits to the use of health technology assessment processes with important implications for policy and practice. The study findings indicate accrued benefits as a result of increased transparency, effective inclusion of stakeholders’ preferences, and value for money in decisions made [44]. A South African study on the application of context-specific ethics frameworks in decision-making revealed that deliberations enhanced appraisal quality and transparency [56]. The impact of implementing health technology assessment decision recommendations on SRHR included workforce strengthening and improved service delivery. However, the non-use of systematic approaches to SRHR resource allocation resulted in inequalities in healthcare access and poor implementation. There is evidence that monitoring and evaluation approaches introduced by research institutions within the advisory committees are likely to be adopted in priority-setting [57].

This review’s novelty of applying the evidence-informed deliberative processes framework on health technology assessment for SRHR priority-setting and benefits package design has limitations, including missing data on key thematic areas for the framework. It’s worth noting that just 6 of the 14 included studies were carried out after the evidence-informed deliberative processes framework was formulated. While it is unlikely that they would have followed the evidence-informed deliberative processes step by step, they would have followed its principles, which have long been established in stakeholder involvement and deliberative processes. In our contextual review of health technology assessment in each study, we did not independently verify with literature or use our prior knowledge of included countries to triangulate any information. As a consequence of the foregoing, the unit of study was each included article and not the country the article was carried in. The study used the face value for the included studies. Furthermore, although desirable, following the evidence-informed deliberative processes framework, this review could not conclusively characterise the performance of SRHR decisions as part of general health benefit package decisions versus specific SHRH package decisions. As a consequence, evidence from this study cannot characterise if there are specific procedural requirements, that SRHR decisions should meet given the characteristics of the interventions, the stakeholders affected by these and the influence of values. Additional limitations include the absence of a quality assessment of the included studies and the use of a single reviewer for the title and abstract screening stage.

## Conclusion

This review has shown potential benefits accrued in applying the evidence-informed deliberative processes framework for health technology assessment to improve stakeholder involvement and deliberations in SRHR priority-setting and benefits package design. The implications of the study findings for policy and practice highlight the need for policymakers and healthcare professionals to prioritise evidence-informed deliberative processes in the design of benefits packages for SRHR in sub-Saharan Africa. The use of the evidence-informed deliberative processes framework for priority-setting is particularly important in SRHR programming and benefits package design to promote equity, efficiency, and legitimacy, as diverse stakeholders with multiple social values are likely to affect the legitimacy of decision recommendations. To the best of our knowledge, this is the first review to systematically evaluate the use of a deliberative framework for health technology assessment in SRHR benefits package design across sub-Saharan Africa.

As a policy recommendation, sub-Saharan African Governments could consider moving towards institutionalizing health technology assessment and generating explicit policy statements and sufficient yearly budgets for its use. There must be deliberate efforts to enhance health technology assessment networking and support for stakeholder capacity strengthening. Specifically, there is a need for enhanced involvement of stakeholders with diverse backgrounds for SRHR benefits package design. Initiating an advisory committee for each decision-making process, including benefits package design and revision and SRHR program-specific interventions, should follow transparent recruitment processes, with clearly defined roles and voting responsibilities. Consensus-building techniques, as identified by the recent deliberation guidance by the Professional Society for Health Economics and Outcomes Research, and Health Technology Assessment International [58], are seen as good practice.

In-country decision criteria for the selection and reimbursement of health technology should be in place to guide the selection of technologies for assessment following the identification and appraisal stages. Furthermore, Identification, selection, scoping, assessment, appraisal, and appeal of SRHR health technologies should follow exhaustive stakeholder involvement within the advisory group and wider population. Guidelines for economic evaluations, socio-cultural, ethics, equity, legal, effectiveness, quality, and safety should be established to support SRHR health technology assessment. There must be an explicit channel to support stakeholder appeals for decision recommendations. As an implication for further research, there is a need to quantify the return on investment for the use of deliberative processes in health technology assessment for SRHR benefits designs. This would require appropriate tools for health technology assessment inputs, outputs, outcomes, and impacts [59]. Development assistance partners should work closely with other stakeholders, including the government, and advance more research and practices to strengthen the legitimacy, accountability, and transparency of these processes.

## Supporting information

S1 File PRISMA checklist. (DOCX)

S2 File Search terms and PubMed search strategy. (DOCX)

S3 File Full list of institutional websites searched. (DOCX)

S4 File Characteristics of included studies. (DOCX)
