# Supplementary material for: Health technology assessment for sexual reproductive health and rights benefits package design in sub-Saharan Africa: A scoping review of evidence-informed deliberative processes
Source: PLoS One. 2024 Jun 27;19(6):e0306042. doi: 10.1371/journal.pone.0306042 (PMC11210850; doi:10.1371/journal.pone.0306042)
Supplement: S2 File — (DOCX) [file pone.0306042.s002.docx]

**Section 1: Search Terms**

The study used the Guttmacher–Lancet Commission proposed comprehensive definition for sexual reproductive health and rights [1] to identify the benefits package for the review. To establish an exhaustive search strategy, the Guttmacher–Lancet Commission package of interventions was mapped to the WHO universal health coverage compendium [2]. This represents 23 interventions in the WHO UHC Compendium for sexual reproductive health and rights and 171 actions. For completeness, we also used additional sources to map the comprehensive sexuality education disease program area [3]. The interventions and actions were used to establish key terms, words and phrases for the search strings, as shown below.

| **Categories** | **Key Terms** | **Words and Phrases** |
| --- | --- | --- |
| Reproductive and Sexual health | Pregnancy and Birth, Labour and childbirth care, Postnatal care, care for newborn complications, Care for postnatal complications of the mother, | **"Fetal auscultation" OR "prostaglandin analogues" OR "Intravenous analgesics" OR uterotonics OR "Routine vaginal delivery" OR partograph OR tocolytics OR uterotonics OR prostaglandin OR "postpartum haemorrhage" OR "antenatal corticosteroids" OR tocolytics OR uterotonics OR vasopressors OR analogues OR Amniotomy OR "Bimanual uterine compression" OR "Caesarean section" OR "laceration repair" OR cephalic OR Hysterectomy OR "Intrauterine tamponade" OR "shoulder dystocia" OR "uterine cavity" OR "vaginal delivery" OR "Vaginal breech" OR "Fetal heartrate" OR "Postnatal care" OR "exclusive breastfeeding" OR "prevention of mother to child transmission" OR hygiene OR handwashing OR vaccinations OR "birth defects" OR "congenital abnormalities" OR "automated auditory brainstem response" OR "otoacoustic emissions" OR "neonatal ophthalmia" OR "vitamin K" OR "postpartum depression" OR "Anti-D immune globulin" OR "neonatal complications" OR "Micronutrient supplementation" OR apnea OR "bronchopulmonary dysplasia" OR "retinopathy of prematurity" OR "hypoxemic encephalopathy" OR "ischaemic encephalopathy" OR "Lumbar puncture" OR "Pulse oximetry" OR laparotomy OR "postnatal complications" OR curettage OR Hysterectomy OR "Urinary catheterization" OR "Uterine artery embolization"** |
|  | Abortion,  Care for complications of abortion and Ectopic pregnancy | **"Abortion related care" OR "Oral analgesics" OR "Oral antimicrobials" OR "Oral progesterone" OR "Oral prostaglandins" OR "Anti-D immunoglobulin" OR "Dilation and evacuation" OR "Electric vacuum aspiration" OR "Manual vacuum aspiration" OR "Female sterilization" OR "complications of abortion" OR "Oral iron supplementation" OR "septic abortion"** OR anaemia OR Blood OR “genital trauma” OR “Ectopic pregnancy” OR “ectopic pregnancy” OR “Anti-D immune globulin” OR antimetabolite OR Salpingectomy |
|  | Contraception and family planning, Management of family planning and contraception | **"family planning" AND Contraception OR Screening OR counseling OR "Laboratory tests" OR nonhormonal OR "short-acting hormonal" OR "long-acting hormonal" OR sterilization OR condoms OR "self-care" OR "Oral hormonal contraceptive" OR Diaphragms OR "emergency contraception" OR "post-abortion contraceptives" OR "Postpartum contraceptives" OR "Injectable hormonal contraceptive" OR "Intrauterine devices" OR "Removal of implant" OR "Subdermal contraceptive implant" OR Vasectomy OR "Vaginal ring"** |
|  | Infertility, prevention of infertility and Diagnosis of infertility | **Infertility OR Cryopreservation OR "ovulation predictor kits" OR Genetic testing OR Hormonal assays OR "ovarian reserve assessment" OR "Semen analysis"** |
|  | Sexual Health, gender non-conforming and gender affirming hormone therapy | **"sexual health and wellbeing" OR "gender norms" OR sexuality OR sexual health OR "age of maturation" OR "gender non-conforming" OR "gender-affirming" OR "Oral hormone therapy" OR "Topical hormone therapy"** |
|  | Female genital mutilation (FGM), Intimate partner and sexual violence | **"Female genital mutilation" OR "De-infibulation" OR "Intimate partner and sexual violence" OR rape OR "mental disorders" OR Psychoeducation OR "post-exposure prophylaxis" OR "emergency contraception" OR "Hepatitis B" OR HPV** |
|  | Comprehensive Sexuality education [3] | **Education AND "Comprehensive Sexuality education" OR "curriculum-based" OR "sexual relationships" OR "sexual and reproductive health" OR "sexuality" OR "gender equality" OR "Young people's rights" OR "Abstinence-plus" OR "Sexuality Education"** |
| Noncommunicable diseases and mental health | Cervical cancer, Early detection, diagnosis and staging of cervical cancer, Management of pre-invasive cervical cancer, Early detection, diagnosis and staging of cervical cancer, Management of cervical cancer, Management of metastatic cervical cancer | **“Cervical Cancer” AND "Prevention of cervical cancer" OR Vaccination OR HPV OR "Visual inspection with acetic acid" OR Cytology OR Biopsy OR Colposcopy OR "Intravenous pyelography" OR Cystoscopy OR Proctoscopy OR "Laparoscopic staging" OR "Cold knife conization" OR "pre-invasive cervical cancer" OR Cryotherapy OR "Thermal ablation" OR "Loop electrical excision procedure" OR "Large loop excision of transformation zone" OR hysterectomy OR "psycho-oncology" OR "pain management" OR Chemotherapy OR "IV analgesics" OR "Modified radical hysterectomy" OR "Radical Trachelectomy" OR incontinence OR lymphedema OR vaginal stenosis OR fistulae OR "Pelvic lymph node dissection" OR "beam radiotherapy" OR Brachytherapy OR colostomy OR nephrostomy OR "metastatic cervical cancer"** |
| Communicable diseases (excluding NTDs) | Prevention of HIV, Management of HIV, Screening and diagnosis of HIV | **HIV AND condoms OR "needle and syringe programmes" OR "opiate substitution therapy" OR "Vaginal microbicide gel" OR "Post-exposure prophylaxis" OR antiretrovirals OR "Pre-exposure prophylaxis" OR "STI screening" OR "Voluntary medical male circumcision" OR "self-collection" OR "HIV testing services" OR "cancer screening" OR gonorrhea OR "hepatitis B" OR "hepatitis C" OR syphilis OR tuberculosis OR "Isoniazid preventive therapy" OR antiretrovirals OR NNRTI OR Pneumococcal OR Influenza OR depression OR "adverse drug reactions" OR "drug interactions" OR chlamydia OR coccidiosis OR "Computed tomography" OR "X-ray" OR "Lumbar puncture" OR fibrates OR "HMG Co-A reductase" OR "cryptococcal meningitis" OR Bronchoscopy OR "fluconazole preemptive therapy" OR "Co-trimoxazole prophylaxis"** |
|  | Sexually transmitted and reproductive tract infections, Prevention of STIs and reproductive tract infections, Screening and diagnosis of STIs and reproductive tract infections | “Sexually transmitted” AND **"STI prevention" OR "reproductive tract infections" OR "safer sex" OR "Condom promotion" OR "partner notification" OR "Post-exposure prophylaxis" OR "Human papillomavirus" OR "voluntary medical male circumcision" OR "Hepatitis B" OR "ophthalmia neonatorum" OR Speculum OR Anoscopic OR Bimanual OR "Lumbar puncture" OR Laparoscopy OR condoms OR "intravenous antimicrobials" OR "Urine culture" OR "Transurethral ultrasound" OR "Urethral swab"** |
| Systematic Decision  Making | Health technology Assessment, Priority Setting and Decision making | “Health technology assessment” OR “decision making” OR “Priority Setting” OR “Evidence-informed decision making” OR “Accountability for Reasonableness” OR “Multi-criteria Decision-Making Analysis” OR “composite league table” OR “Resource Allocation” |
| Health Benefits Packages | Essential package of health services and health benefits packages | **“Essential package of health services" OR "health benefits package" OR "essential health care package" OR "essential health services" OR Package OR "Service package"** |
| Regions | Sub-Saharan Countries | **Sub-Saharan Africa OR West Africa OR Southern Africa OR central Africa OR East Africa OR Djibouti OR Seychelles OR DR Congo OR Comoros OR Togo OR Sierra Leone OR Libya OR Tanzania OR South Africa OR Cabo Verde OR Congo OR Kenya OR Liberia OR Central African Republic OR Mauritania OR Uganda OR Algeria OR Sudan OR Eritrea OR Angola OR Mozambique OR Ghana OR Madagascar OR Cameroon OR Côte d'Ivoire OR Namibia Niger OR Gambia OR Botswana OR Gabon OR Sao Tome & Principe OR Lesotho OR Burkina Faso OR Nigeria OR Mali OR Guinea-Bissau OR Malawi OR Zambia OR Senegal OR Chad OR Somalia OR Zimbabwe OR Equatorial Guinea OR Guinea OR Rwanda OR Mauritius OR Benin OR Burundi OR Eswatini OR Ethiopia OR South Sudan** |

**Section 2: PubMed search strategy**

| **16** | (((((((((((("Fetal auscultation" OR "prostaglandin analogues" OR "Intravenous analgesics" OR uterotonics OR "Routine vaginal delivery" OR partograph OR tocolytics OR uterotonics OR prostaglandin OR "postpartum haemorrhage" OR "antenatal corticosteroids" OR tocolytics OR uterotonics OR vasopressors OR analogues OR Amniotomy OR "Bimanual uterine compression" OR "Caesarean section" OR "laceration repair" OR cephalic OR Hysterectomy OR "Intrauterine tamponade" OR "shoulder dystocia" OR "uterine cavity" OR "vaginal delivery" OR "Vaginal breech" OR "Fetal heartrate" OR "Postnatal care" OR "exclusive breastfeeding" OR "prevention of mother to child transmission" OR hygiene OR handwashing OR vaccinations OR "birth defects" OR "congenital abnormalities" OR "automated auditory brainstem response" OR "otoacoustic emissions" OR "neonatal ophthalmia" OR "vitamin K" OR "postpartum depression" OR "Anti-D immune globulin" OR "neonatal complications" OR "Micronutrient supplementation" OR apnea OR "bronchopulmonary dysplasia" OR "retinopathy of prematurity" OR "hypoxemic encephalopathy" OR "ischaemic encephalopathy" OR "Lumbar puncture" OR "Pulse oximetry" OR laparotomy OR "postnatal complications" OR curettage OR Hysterectomy OR "Urinary catheterization" OR "Uterine artery embolization") OR ("Abortion related care" OR "Oral analgesics" OR "Oral antimicrobials" OR "Oral progesterone" OR "Oral prostaglandins" OR "Anti-D immunoglobulin" OR "Dilation and evacuation" OR "Electric vacuum aspiration" OR "Manual vacuum aspiration" OR "Female sterilization" OR "complications of abortion" OR "Oral iron supplementation" OR "septic abortion" OR anaemia OR Blood OR "genital trauma" OR "Ectopic pregnancy" OR "ectopic pregnancy" OR "Anti-D immune globulin" OR antimetabolite OR Salpingectomy)) OR ("family planning" AND Contraception OR Screening OR counseling OR "Laboratory tests" OR nonhormonal OR "short-acting hormonal" OR "long-acting hormonal" OR sterilization OR condoms OR "self-care" OR "Oral hormonal contraceptive" OR Diaphragms OR "emergency contraception" OR "post-abortion contraceptives" OR "Postpartum contraceptives" OR "Injectable hormonal contraceptive" OR "Intrauterine devices" OR "Removal of implant" OR "Subdermal contraceptive implant" OR Vasectomy OR "Vaginal ring")) OR (Infertility OR Cryopreservation OR "ovulation predictor kits" OR Genetic testing OR Hormonal assays OR "ovarian reserve assessment" OR "Semen analysis")) OR ("sexual health and wellbeing" OR "gender norms" OR sexuality OR sexual health OR "age of maturation" OR "gender non-conforming" OR "gender-affirming" OR "Oral hormone therapy" OR "Topical hormone therapy")) OR ("Female genital mutilation" OR "De-infibulation" OR "Intimate partner and sexual violence" OR rape OR "mental disorders" OR Psychoeducation OR "post-exposure prophylaxis" OR "emergency contraception" OR "Hepatitis B" OR HPV)) OR (Education AND "Comprehensive Sexuality education" OR "curriculum-based" OR "sexual relationships" OR "sexual and reproductive health" OR "sexuality" OR "gender equality" OR "Young people's rights" OR "Abstinence-plus" OR "Sexuality Education")) OR ("Cervical Cancer" AND "Prevention of cervical cancer" OR Vaccination OR HPV OR "Visual inspection with acetic acid" OR Cytology OR Biopsy OR Colposcopy OR "Intravenous pyelography" OR Cystoscopy OR Proctoscopy OR "Laparoscopic staging" OR "Cold knife conization" OR "pre-invasive cervical cancer" OR Cryotherapy OR "Thermal ablation" OR "Loop electrical excision procedure" OR "Large loop excision of transformation zone" OR hysterectomy OR "psycho-oncology" OR "pain management" OR Chemotherapy OR "IV analgesics" OR "Modified radical hysterectomy" OR "Radical Trachelectomy" OR incontinence OR lymphedema OR vaginal stenosis OR fistulae OR "Pelvic lymph node dissection" OR "beam radiotherapy" OR Brachytherapy OR colostomy OR nephrostomy OR "metastatic cervical cancer")) OR (HIV AND condoms OR "needle and syringe programmes" OR "opiate substitution therapy" OR "Vaginal microbicide gel" OR "Post-exposure prophylaxis" OR antiretrovirals OR "Pre-exposure prophylaxis" OR "STI screening" OR "Voluntary medical male circumcision" OR "self-collection" OR "HIV testing services" OR "cancer screening" OR gonorrhea OR "hepatitis B" OR "hepatitis C" OR syphilis OR tuberculosis OR "Isoniazid preventive therapy" OR antiretrovirals OR NNRTI OR Pneumococcal OR Influenza OR depression OR "adverse drug reactions" OR "drug interactions" OR chlamydia OR coccidiosis OR "Computed tomography" OR "X-ray" OR "Lumbar puncture" OR fibrates OR "HMG Co-A reductase" OR "cryptococcal meningitis" OR Bronchoscopy OR "fluconazole preemptive therapy" OR "Co-trimoxazole prophylaxis")) OR ("Sexually transmitted" AND "STI prevention" OR "reproductive tract infections" OR "safer sex" OR "Condom promotion" OR "partner notification" OR "Post-exposure prophylaxis" OR "Human papillomavirus" OR "voluntary medical male circumcision" OR "Hepatitis B" OR "ophthalmia neonatorum" OR Speculum OR Anoscopic OR Bimanual OR "Lumbar puncture" OR Laparoscopy OR condoms OR "intravenous antimicrobials" OR "Urine culture" OR "Transurethral ultrasound" OR "Urethral swab")) AND ("Health technology assessment" OR "decision making" OR "Priority Setting" OR "Evidence-informed decision making" OR "Accountability for Reasonableness" OR "Multi-criteria Decision Making Analysis" OR "composite league table" OR "Resource Allocation")) AND ("Essential package of health services" OR "health benefits package" OR "essential health care package" OR "essential health services" OR Package OR "Service package")) AND (Sub-Saharan Africa OR West Africa OR Southern Africa OR central Africa OR East Africa OR Djibouti OR Seychelles OR DR Congo OR Comoros OR Togo OR Sierra Leone OR Libya OR Tanzania OR South Africa OR Cabo Verde OR Congo OR Kenya OR Liberia OR Central African Republic OR Mauritania OR Uganda OR Algeria OR Sudan OR Eritrea OR Angola OR Mozambique OR Ghana OR Madagascar OR Cameroon OR Côte d'Ivoire OR Namibia Niger OR Gambia OR Botswana OR Gabon OR Sao Tome & Principe OR Lesotho OR Burkina Faso OR Nigeria OR Mali OR Guinea-Bissau OR Malawi OR Zambia OR Senegal OR Chad OR Somalia OR Zimbabwe OR Equatorial Guinea OR Guinea OR Rwanda OR Mauritius OR Benin OR Burundi OR Eswatini OR Ethiopia OR South Sudan) |
| --- | --- |
| **15** | (((((((((((("Fetal auscultation" OR "prostaglandin analogues" OR "Intravenous analgesics" OR uterotonics OR "Routine vaginal delivery" OR partograph OR tocolytics OR uterotonics OR prostaglandin OR "postpartum haemorrhage" OR "antenatal corticosteroids" OR tocolytics OR uterotonics OR vasopressors OR analogues OR Amniotomy OR "Bimanual uterine compression" OR "Caesarean section" OR "laceration repair" OR cephalic OR Hysterectomy OR "Intrauterine tamponade" OR "shoulder dystocia" OR "uterine cavity" OR "vaginal delivery" OR "Vaginal breech" OR "Fetal heartrate" OR "Postnatal care" OR "exclusive breastfeeding" OR "prevention of mother to child transmission" OR hygiene OR handwashing OR vaccinations OR "birth defects" OR "congenital abnormalities" OR "automated auditory brainstem response" OR "otoacoustic emissions" OR "neonatal ophthalmia" OR "vitamin K" OR "postpartum depression" OR "Anti-D immune globulin" OR "neonatal complications" OR "Micronutrient supplementation" OR apnea OR "bronchopulmonary dysplasia" OR "retinopathy of prematurity" OR "hypoxemic encephalopathy" OR "ischaemic encephalopathy" OR "Lumbar puncture" OR "Pulse oximetry" OR laparotomy OR "postnatal complications" OR curettage OR Hysterectomy OR "Urinary catheterization" OR "Uterine artery embolization") OR ("Abortion related care" OR "Oral analgesics" OR "Oral antimicrobials" OR "Oral progesterone" OR "Oral prostaglandins" OR "Anti-D immunoglobulin" OR "Dilation and evacuation" OR "Electric vacuum aspiration" OR "Manual vacuum aspiration" OR "Female sterilization" OR "complications of abortion" OR "Oral iron supplementation" OR "septic abortion" OR anaemia OR Blood OR "genital trauma" OR "Ectopic pregnancy" OR "ectopic pregnancy" OR "Anti-D immune globulin" OR antimetabolite OR Salpingectomy)) OR ("family planning" AND Contraception OR Screening OR counseling OR "Laboratory tests" OR nonhormonal OR "short-acting hormonal" OR "long-acting hormonal" OR sterilization OR condoms OR "self-care" OR "Oral hormonal contraceptive" OR Diaphragms OR "emergency contraception" OR "post-abortion contraceptives" OR "Postpartum contraceptives" OR "Injectable hormonal contraceptive" OR "Intrauterine devices" OR "Removal of implant" OR "Subdermal contraceptive implant" OR Vasectomy OR "Vaginal ring")) OR (Infertility OR Cryopreservation OR "ovulation predictor kits" OR Genetic testing OR Hormonal assays OR "ovarian reserve assessment" OR "Semen analysis")) OR ("sexual health and wellbeing" OR "gender norms" OR sexuality OR sexual health OR "age of maturation" OR "gender non-conforming" OR "gender-affirming" OR "Oral hormone therapy" OR "Topical hormone therapy")) OR ("Female genital mutilation" OR "De-infibulation" OR "Intimate partner and sexual violence" OR rape OR "mental disorders" OR Psychoeducation OR "post-exposure prophylaxis" OR "emergency contraception" OR "Hepatitis B" OR HPV)) OR (Education AND "Comprehensive Sexuality education" OR "curriculum-based" OR "sexual relationships" OR "sexual and reproductive health" OR "sexuality" OR "gender equality" OR "Young people's rights" OR "Abstinence-plus" OR "Sexuality Education")) OR ("Cervical Cancer" AND "Prevention of cervical cancer" OR Vaccination OR HPV OR "Visual inspection with acetic acid" OR Cytology OR Biopsy OR Colposcopy OR "Intravenous pyelography" OR Cystoscopy OR Proctoscopy OR "Laparoscopic staging" OR "Cold knife conization" OR "pre-invasive cervical cancer" OR Cryotherapy OR "Thermal ablation" OR "Loop electrical excision procedure" OR "Large loop excision of transformation zone" OR hysterectomy OR "psycho-oncology" OR "pain management" OR Chemotherapy OR "IV analgesics" OR "Modified radical hysterectomy" OR "Radical Trachelectomy" OR incontinence OR lymphedema OR vaginal stenosis OR fistulae OR "Pelvic lymph node dissection" OR "beam radiotherapy" OR Brachytherapy OR colostomy OR nephrostomy OR "metastatic cervical cancer")) OR (HIV AND condoms OR "needle and syringe programmes" OR "opiate substitution therapy" OR "Vaginal microbicide gel" OR "Post-exposure prophylaxis" OR antiretrovirals OR "Pre-exposure prophylaxis" OR "STI screening" OR "Voluntary medical male circumcision" OR "self-collection" OR "HIV testing services" OR "cancer screening" OR gonorrhea OR "hepatitis B" OR "hepatitis C" OR syphilis OR tuberculosis OR "Isoniazid preventive therapy" OR antiretrovirals OR NNRTI OR Pneumococcal OR Influenza OR depression OR "adverse drug reactions" OR "drug interactions" OR chlamydia OR coccidiosis OR "Computed tomography" OR "X-ray" OR "Lumbar puncture" OR fibrates OR "HMG Co-A reductase" OR "cryptococcal meningitis" OR Bronchoscopy OR "fluconazole preemptive therapy" OR "Co-trimoxazole prophylaxis")) OR ("Sexually transmitted" AND "STI prevention" OR "reproductive tract infections" OR "safer sex" OR "Condom promotion" OR "partner notification" OR "Post-exposure prophylaxis" OR "Human papillomavirus" OR "voluntary medical male circumcision" OR "Hepatitis B" OR "ophthalmia neonatorum" OR Speculum OR Anoscopic OR Bimanual OR "Lumbar puncture" OR Laparoscopy OR condoms OR "intravenous antimicrobials" OR "Urine culture" OR "Transurethral ultrasound" OR "Urethral swab")) AND ("Health technology assessment" OR "decision making" OR "Priority Setting" OR "Evidence-informed decision making" OR "Accountability for Reasonableness" OR "Multi-criteria Decision Making Analysis" OR "composite league table" OR "Resource Allocation")) AND ("Essential package of health services" OR "health benefits package" OR "essential health care package" OR "essential health services" OR Package OR "Service package")) AND (Sub-Saharan Africa OR West Africa OR Southern Africa OR central Africa OR East Africa OR Djibouti OR Seychelles OR DR Congo OR Comoros OR Togo OR Sierra Leone OR Libya OR Tanzania OR South Africa OR Cabo Verde OR Congo OR Kenya OR Liberia OR Central African Republic OR Mauritania OR Uganda OR Algeria OR Sudan OR Eritrea OR Angola OR Mozambique OR Ghana OR Madagascar OR Cameroon OR Côte d'Ivoire OR Namibia Niger OR Gambia OR Botswana OR Gabon OR Sao Tome & Principe OR Lesotho OR Burkina Faso OR Nigeria OR Mali OR Guinea-Bissau OR Malawi OR Zambia OR Senegal OR Chad OR Somalia OR Zimbabwe OR Equatorial Guinea OR Guinea OR Rwanda OR Mauritius OR Benin OR Burundi OR Eswatini OR Ethiopia OR South Sudan) |
| **14** | (((((((((((("Fetal auscultation" OR "prostaglandin analogues" OR "Intravenous analgesics" OR uterotonics OR "Routine vaginal delivery" OR partograph OR tocolytics OR uterotonics OR prostaglandin OR "postpartum haemorrhage" OR "antenatal corticosteroids" OR tocolytics OR uterotonics OR vasopressors OR analogues OR Amniotomy OR "Bimanual uterine compression" OR "Caesarean section" OR "laceration repair" OR cephalic OR Hysterectomy OR "Intrauterine tamponade" OR "shoulder dystocia" OR "uterine cavity" OR "vaginal delivery" OR "Vaginal breech" OR "Fetal heartrate" OR "Postnatal care" OR "exclusive breastfeeding" OR "prevention of mother to child transmission" OR hygiene OR handwashing OR vaccinations OR "birth defects" OR "congenital abnormalities" OR "automated auditory brainstem response" OR "otoacoustic emissions" OR "neonatal ophthalmia" OR "vitamin K" OR "postpartum depression" OR "Anti-D immune globulin" OR "neonatal complications" OR "Micronutrient supplementation" OR apnea OR "bronchopulmonary dysplasia" OR "retinopathy of prematurity" OR "hypoxemic encephalopathy" OR "ischaemic encephalopathy" OR "Lumbar puncture" OR "Pulse oximetry" OR laparotomy OR "postnatal complications" OR curettage OR Hysterectomy OR "Urinary catheterization" OR "Uterine artery embolization") OR ("Abortion related care" OR "Oral analgesics" OR "Oral antimicrobials" OR "Oral progesterone" OR "Oral prostaglandins" OR "Anti-D immunoglobulin" OR "Dilation and evacuation" OR "Electric vacuum aspiration" OR "Manual vacuum aspiration" OR "Female sterilization" OR "complications of abortion" OR "Oral iron supplementation" OR "septic abortion" OR anaemia OR Blood OR "genital trauma" OR "Ectopic pregnancy" OR "ectopic pregnancy" OR "Anti-D immune globulin" OR antimetabolite OR Salpingectomy)) OR ("family planning" AND Contraception OR Screening OR counseling OR "Laboratory tests" OR nonhormonal OR "short-acting hormonal" OR "long-acting hormonal" OR sterilization OR condoms OR "self-care" OR "Oral hormonal contraceptive" OR Diaphragms OR "emergency contraception" OR "post-abortion contraceptives" OR "Postpartum contraceptives" OR "Injectable hormonal contraceptive" OR "Intrauterine devices" OR "Removal of implant" OR "Subdermal contraceptive implant" OR Vasectomy OR "Vaginal ring")) OR (Infertility OR Cryopreservation OR "ovulation predictor kits" OR Genetic testing OR Hormonal assays OR "ovarian reserve assessment" OR "Semen analysis")) OR ("sexual health and wellbeing" OR "gender norms" OR sexuality OR sexual health OR "age of maturation" OR "gender non-conforming" OR "gender-affirming" OR "Oral hormone therapy" OR "Topical hormone therapy")) OR ("Female genital mutilation" OR "De-infibulation" OR "Intimate partner and sexual violence" OR rape OR "mental disorders" OR Psychoeducation OR "post-exposure prophylaxis" OR "emergency contraception" OR "Hepatitis B" OR HPV)) OR (Education AND "Comprehensive Sexuality education" OR "curriculum-based" OR "sexual relationships" OR "sexual and reproductive health" OR "sexuality" OR "gender equality" OR "Young people's rights" OR "Abstinence-plus" OR "Sexuality Education")) OR ("Cervical Cancer" AND "Prevention of cervical cancer" OR Vaccination OR HPV OR "Visual inspection with acetic acid" OR Cytology OR Biopsy OR Colposcopy OR "Intravenous pyelography" OR Cystoscopy OR Proctoscopy OR "Laparoscopic staging" OR "Cold knife conization" OR "pre-invasive cervical cancer" OR Cryotherapy OR "Thermal ablation" OR "Loop electrical excision procedure" OR "Large loop excision of transformation zone" OR hysterectomy OR "psycho-oncology" OR "pain management" OR Chemotherapy OR "IV analgesics" OR "Modified radical hysterectomy" OR "Radical Trachelectomy" OR incontinence OR lymphedema OR vaginal stenosis OR fistulae OR "Pelvic lymph node dissection" OR "beam radiotherapy" OR Brachytherapy OR colostomy OR nephrostomy OR "metastatic cervical cancer")) OR (HIV AND condoms OR "needle and syringe programmes" OR "opiate substitution therapy" OR "Vaginal microbicide gel" OR "Post-exposure prophylaxis" OR antiretrovirals OR "Pre-exposure prophylaxis" OR "STI screening" OR "Voluntary medical male circumcision" OR "self-collection" OR "HIV testing services" OR "cancer screening" OR gonorrhea OR "hepatitis B" OR "hepatitis C" OR syphilis OR tuberculosis OR "Isoniazid preventive therapy" OR antiretrovirals OR NNRTI OR Pneumococcal OR Influenza OR depression OR "adverse drug reactions" OR "drug interactions" OR chlamydia OR coccidiosis OR "Computed tomography" OR "X-ray" OR "Lumbar puncture" OR fibrates OR "HMG Co-A reductase" OR "cryptococcal meningitis" OR Bronchoscopy OR "fluconazole preemptive therapy" OR "Co-trimoxazole prophylaxis")) OR ("Sexually transmitted" AND "STI prevention" OR "reproductive tract infections" OR "safer sex" OR "Condom promotion" OR "partner notification" OR "Post-exposure prophylaxis" OR "Human papillomavirus" OR "voluntary medical male circumcision" OR "Hepatitis B" OR "ophthalmia neonatorum" OR Speculum OR Anoscopic OR Bimanual OR "Lumbar puncture" OR Laparoscopy OR condoms OR "intravenous antimicrobials" OR "Urine culture" OR "Transurethral ultrasound" OR "Urethral swab")) AND ("Health technology assessment" OR "decision making" OR "Priority Setting" OR "Evidence-informed decision making" OR "Accountability for Reasonableness" OR "Multi-criteria Decision Making Analysis" OR "composite league table" OR "Resource Allocation")) AND ("Essential package of health services" OR "health benefits package" OR "essential health care package" OR "essential health services" OR Package OR "Service package")) AND (Sub-Saharan Africa OR West Africa OR Southern Africa OR central Africa OR East Africa OR Djibouti OR Seychelles OR DR Congo OR Comoros OR Togo OR Sierra Leone OR Libya OR Tanzania OR South Africa OR Cabo Verde OR Congo OR Kenya OR Liberia OR Central African Republic OR Mauritania OR Uganda OR Algeria OR Sudan OR Eritrea OR Angola OR Mozambique OR Ghana OR Madagascar OR Cameroon OR Côte d'Ivoire OR Namibia Niger OR Gambia OR Botswana OR Gabon OR Sao Tome & Principe OR Lesotho OR Burkina Faso OR Nigeria OR Mali OR Guinea-Bissau OR Malawi OR Zambia OR Senegal OR Chad OR Somalia OR Zimbabwe OR Equatorial Guinea OR Guinea OR Rwanda OR Mauritius OR Benin OR Burundi OR Eswatini OR Ethiopia OR South Sudan) |
| **13** | Sub-Saharan Africa OR West Africa OR Southern Africa OR central Africa OR East Africa OR Djibouti OR Seychelles OR DR Congo OR Comoros OR Togo OR Sierra Leone OR Libya OR Tanzania OR South Africa OR Cabo Verde OR Congo OR Kenya OR Liberia OR Central African Republic OR Mauritania OR Uganda OR Algeria OR Sudan OR Eritrea OR Angola OR Mozambique OR Ghana OR Madagascar OR Cameroon OR Côte d'Ivoire OR Namibia Niger OR Gambia OR Botswana OR Gabon OR Sao Tome & Principe OR Lesotho OR Burkina Faso OR Nigeria OR Mali OR Guinea-Bissau OR Malawi OR Zambia OR Senegal OR Chad OR Somalia OR Zimbabwe OR Equatorial Guinea OR Guinea OR Rwanda OR Mauritius OR Benin OR Burundi OR Eswatini OR Ethiopia OR South Sudan |
| **12** | "Essential package of health services" OR "health benefits package" OR "essential health care package" OR "essential health services" OR Package OR "Service package" |
| **11** | "Health technology assessment" OR "decision making" OR "Priority Setting" OR "Evidence-informed decision making" OR "Accountability for Reasonableness" OR "Multi-criteria Decision Making Analysis" OR "composite league table" OR "Resource Allocation" |
| **10** | "Sexually transmitted" AND "STI prevention" OR "reproductive tract infections" OR "safer sex" OR "Condom promotion" OR "partner notification" OR "Post-exposure prophylaxis" OR "Human papillomavirus" OR "voluntary medical male circumcision" OR "Hepatitis B" OR "ophthalmia neonatorum" OR Speculum OR Anoscopic OR Bimanual OR "Lumbar puncture" OR Laparoscopy OR condoms OR "intravenous antimicrobials" OR "Urine culture" OR "Transurethral ultrasound" OR "Urethral swab" |
| **9** | HIV AND condoms OR "needle and syringe programmes" OR "opiate substitution therapy" OR "Vaginal microbicide gel" OR "Post-exposure prophylaxis" OR antiretrovirals OR "Pre-exposure prophylaxis" OR "STI screening" OR "Voluntary medical male circumcision" OR "self-collection" OR "HIV testing services" OR "cancer screening" OR gonorrhea OR "hepatitis B" OR "hepatitis C" OR syphilis OR tuberculosis OR "Isoniazid preventive therapy" OR antiretrovirals OR NNRTI OR Pneumococcal OR Influenza OR depression OR "adverse drug reactions" OR "drug interactions" OR chlamydia OR coccidiosis OR "Computed tomography" OR "X-ray" OR "Lumbar puncture" OR fibrates OR "HMG Co-A reductase" OR "cryptococcal meningitis" OR Bronchoscopy OR "fluconazole preemptive therapy" OR "Co-trimoxazole prophylaxis" |
| **8** | "Cervical Cancer" AND "Prevention of cervical cancer" OR Vaccination OR HPV OR "Visual inspection with acetic acid" OR Cytology OR Biopsy OR Colposcopy OR "Intravenous pyelography" OR Cystoscopy OR Proctoscopy OR "Laparoscopic staging" OR "Cold knife conization" OR "pre-invasive cervical cancer" OR Cryotherapy OR "Thermal ablation" OR "Loop electrical excision procedure" OR "Large loop excision of transformation zone" OR hysterectomy OR "psycho-oncology" OR "pain management" OR Chemotherapy OR "IV analgesics" OR "Modified radical hysterectomy" OR "Radical Trachelectomy" OR incontinence OR lymphedema OR vaginal stenosis OR fistulae OR "Pelvic lymph node dissection" OR "beam radiotherapy" OR Brachytherapy OR colostomy OR nephrostomy OR "metastatic cervical cancer" |
| **7** | Education AND "Comprehensive Sexuality education" OR "curriculum-based" OR "sexual relationships" OR "sexual and reproductive health" OR "sexuality" OR "gender equality" OR "Young people's rights" OR "Abstinence-plus" OR "Sexuality Education" |
| **6** | "Female genital mutilation" OR "De-infibulation" OR "Intimate partner and sexual violence" OR rape OR "mental disorders" OR Psychoeducation OR "post-exposure prophylaxis" OR "emergency contraception" OR "Hepatitis B" OR HPV |
| **5** | "sexual health and wellbeing" OR "gender norms" OR sexuality OR sexual health OR "age of maturation" OR "gender non-conforming" OR "gender-affirming" OR "Oral hormone therapy" OR "Topical hormone therapy" |
| **4** | Infertility OR Cryopreservation OR "ovulation predictor kits" OR Genetic testing OR Hormonal assays OR "ovarian reserve assessment" OR "Semen analysis" |
| **3** | "family planning" AND Contraception OR Screening OR counseling OR "Laboratory tests" OR nonhormonal OR "short-acting hormonal" OR "long-acting hormonal" OR sterilization OR condoms OR "self-care" OR "Oral hormonal contraceptive" OR Diaphragms OR "emergency contraception" OR "post-abortion contraceptives" OR "Postpartum contraceptives" OR "Injectable hormonal contraceptive" OR "Intrauterine devices" OR "Removal of implant" OR "Subdermal contraceptive implant" OR Vasectomy OR "Vaginal ring" |
| **2** | "Abortion related care" OR "Oral analgesics" OR "Oral antimicrobials" OR "Oral progesterone" OR "Oral prostaglandins" OR "Anti-D immunoglobulin" OR "Dilation and evacuation" OR "Electric vacuum aspiration" OR "Manual vacuum aspiration" OR "Female sterilization" OR "complications of abortion" OR "Oral iron supplementation" OR "septic abortion" OR anaemia OR Blood OR "genital trauma" OR "Ectopic pregnancy" OR "ectopic pregnancy" OR "Anti-D immune globulin" OR antimetabolite OR Salpingectomy |
| **1** | "Fetal auscultation" OR "prostaglandin analogues" OR "Intravenous analgesics" OR uterotonics OR "Routine vaginal delivery" OR partograph OR tocolytics OR uterotonics OR prostaglandin OR "postpartum haemorrhage" OR "antenatal corticosteroids" OR tocolytics OR uterotonics OR vasopressors OR analogues OR Amniotomy OR "Bimanual uterine compression" OR "Caesarean section" OR "laceration repair" OR cephalic OR Hysterectomy OR "Intrauterine tamponade" OR "shoulder dystocia" OR "uterine cavity" OR "vaginal delivery" OR "Vaginal breech" OR "Fetal heartrate" OR "Postnatal care" OR "exclusive breastfeeding" OR "prevention of mother to child transmission" OR hygiene OR handwashing OR vaccinations OR "birth defects" OR "congenital abnormalities" OR "automated auditory brainstem response" OR "otoacoustic emissions" OR "neonatal ophthalmia" OR "vitamin K" OR "postpartum depression" OR "Anti-D immune globulin" OR "neonatal complications" OR "Micronutrient supplementation" OR apnea OR "bronchopulmonary dysplasia" OR "retinopathy of prematurity" OR "hypoxemic encephalopathy" OR "ischaemic encephalopathy" OR "Lumbar puncture" OR "Pulse oximetry" OR laparotomy OR "postnatal complications" OR curettage OR Hysterectomy OR "Urinary catheterization" OR "Uterine artery embolization" |

**Works Cited**

1. Starrs AM, Ezeh AC, Barker G, Basu A, Bertrand JT, Blum R, et al. Accelerate progress—sexual and reproductive health and rights for all: report of the Guttmacher–Lancet Commission. The Lancet. 2018;391: 2642–2692. doi:10.1016/S0140-6736(18)30293-9

2. WHO. UHC Compendium – Repository of interventions for Universal Health Coverage. 2021. Available: https://www.who.int/universal-health-coverage/compendium/interventions-by-programme-area

3. Miedema E, Le Mat MLJ, Hague F. But is it Comprehensive? Unpacking the ‘comprehensive’ in comprehensive sexuality education. Health Education Journal. 2020;79: 747–762. doi:10.1177/0017896920915960
