# Supplementary material for: Health technology assessment for sexual reproductive health and rights benefits package design in sub-Saharan Africa: A scoping review of evidence-informed deliberative processes
Source: PLoS One. 2024 Jun 27;19(6):e0306042. doi: 10.1371/journal.pone.0306042 (PMC11210850; doi:10.1371/journal.pone.0306042)
Supplement: S3 File — (DOCX) [file pone.0306042.s003.docx]

**Full List of Institutional Websites Searched**

| **SN** | **Organisation** | **Website** |
| --- | --- | --- |
| 1 | Primary Health care Performance Initiative | improvingphc.org |
| 2 | World Health Organisation | who.int |
| 3 | United National Population Fund | unfpa.org |
| 4 | Results for Development | r4d.org |
| 5 | The Global Health Network | tghn.org |
| 6 | Thanzi la Onse | thanzi.org |
| 7 | Child Acute Illness & Nutrition Network | chain.tghn.org |
| 8 | Sexual Reproductive Health and Universal Health Coverage | learn-uhc.srhr.org |
| 9 | Swedish Government Agency for Development Cooperation | sida.se |
