# Supplementary material for: Health technology assessment for sexual reproductive health and rights benefits package design in sub-Saharan Africa: A scoping review of evidence-informed deliberative processes
Source: PLoS One. 2024 Jun 27;19(6):e0306042. doi: 10.1371/journal.pone.0306042 (PMC11210850; doi:10.1371/journal.pone.0306042)
Supplement: S4 File — (DOCX) [file pone.0306042.s004.docx]

**Table 1. Characteristics of Included Studies**

| **Included Study** | **Country of Study** | **SRHR Intervention** | **Research Methods** | **Advisory Committee Assessed** | **Development Assistance Partners** | **HTA Agency** | **HTA Funding** |
| --- | --- | --- | --- | --- | --- | --- | --- |
| Fetters et al., 2017.  Moving from legality to reality: how medical abortion methods were introduced with implementation science in Zambia | Zambia | SRHR Program: Abortion Care | Literature review and cross-sectional survey | Assessment Team | IPAS, WHO | N | N |
| Jackson et al., 2011.  A strategic Assessment of Unsafe Abortion in Malawi | Malawi | SRHR Program: Abortion Care | Literature review and interviews | Assessment Team | UNDP, UNFPA, WHO, World Bank, IPAS | N | N |
| Widdig et al., 2022.  The political economy of priority-setting for health in South Sudan: a case study of the Health Pooled Fund | South Sudan | SRHR package in EPHS | Document review, interviews, and an examination of service delivery | Fund Management Agency | UNICEF, World Food Program, UNFPA, USAID, DFID, GAVI | N | N |
| Mayhew, 2004.  Sexual and reproductive health: challenges for priority-setting in Ghana's health reforms | Ghana | SRHR package in EPHS | Interviews and document review | Committee | USAID, UNFPA, WHO, World Bank, UNICEF | N | N |
| Murphy et al., 2021.  Role of data from cost and other economic analyses in healthcare decision-making for HIV, TB, and sexual/reproductive health programs in South Africa | South Africa | SRHR package in EPHS | Interviews | Committee | PEPFAR, WHO | N | N |
| Eregata et al., 2020.  Revision of the Ethiopian Essential Health Service Package: An Explication of the Process and Methods Used | Ethiopia | SRHR package in EPHS | Qualitative analysis of secondary data on implementation | Technical Working Group | WHO | N | N |
| Owino et al., 2020.  The missing link in Kenya's universal health coverage experiment: a preventive and promotive approach to SRHR | Kenya | SRHR package in EPHS | Qualitative analysis of secondary data on implementation | Advisory Panel | WHO | N | N |
| Memirie et al., 2022.  Addressing the Impact of Noncommunicable Diseases and Injuries (NCDIs) in Ethiopia: Findings and Recommendations from the Ethiopia NCDI Commission | Ethiopia | SRHR Program: Cervical Cancer | A systematic review and quantitative analysis of secondary data | Commission | NDC Synergies at Partners of Health | N | N |
| Davis et al., 2013.  Adaptation of a general primary care package for HIV-infected adults to an HIV centre setting in Gaborone, Botswana | Botswana | SRHR Program: HIV/AIDS | Literature review and quantitative analysis of secondary data | ___ | Baylor Children's Clinical Centre of Excellence | N | N |
| Chitama et al., 2011.  From papers to practices: district-level priority setting processes and criteria for family planning, maternal, newborn and child health interventions in Tanzania | Tanzania | SRHR Program: Family Planning | Nominal group technique, interviews, and literature review | Management Team | Health Basket Funding | N | N |
| Kamuzora et al., 2013.  Promoting community participation in priority setting in district health systems: experiences from Mbarali district, Tanzania | Tanzania | SRHR Program: HIV/AIDS | Interview and qualitative analysis of secondary data | Management Team | European Union | N | N |
| Partnership for Maternal, Newborn & Child Health, 2016.  Case Study Malawi | Malawi | SRHR package in EPHS | Qualitative analysis of secondary data | Technical Working Group | SIDA, Gavi, Global Fund, Global Financing Facility, PMNCH | N | N |
| Kapiriri et al., 2019.  Beyond cost-effectiveness, morbidity and mortality: a comprehensive evaluation of priority setting for HIV programming in Uganda | Uganda | SRHR Program: HIV/AIDS | Interviews and document review | Commission | PEPFAR, Global Fund, USAID, | N | N |
| Chi and Regan 2021.  Investigating the Inclusion of Vertical Programmes in Health Benefits Packages: A case study of Zambia | Zambia | SRHR package in EPHS | Literature review and interviews | Steering Committee | Global Fund, Global Financing Facility, WHO, World Bank | N | N |
| Notes:  Yes = Y, No = N  HTA Agency: based on the given study information about the country, indicates whether there is an established HTA agency  HTA Funding: based on the given study information about the country, indicates whether there is the availability of yearly public funding for HTA | | | | | | | |
